# Subducted organic matter buffered by marine carbonate rules the carbon isotopic signature of arc emissions

**DOI:** 10.1038/s41467-022-30421-5

**Published:** 2022-05-25

**Authors:** S. Tumiati, S. Recchia, L. Remusat, C. Tiraboschi, D. A. Sverjensky, C. E. Manning, A. Vitale Brovarone, A. Boutier, D. Spanu, S. Poli

**Affiliations:** 1 Dipartimento di Scienze della Terra, Università degli Studi di Milano, via Mangiagalli 34, I-20133 Milano, Italy; 2 Dipartimento di Scienza e Alta Tecnologia, Università degli Studi dell’Insubria, via Valleggio 11, I-22100 Como, Italy; 3 Institut de Minéralogie, de Physique des Matériaux, et de Cosmochimie (IMPMC), Sorbonne Universités – UPMC, UMR CNRS, 7590, Muséum National d’Histoire Naturelle, IRD UMR 206, F-75005 Paris, France; 4 Institut für Mineralogie, Universität Münster, Correnstrasse 24, 48149 Münster, Germany; 5 Department of Earth & Planetary Sciences, Johns Hopkins University, Baltimore, MD 21218 USA; 6 Department of Earth, Planetary and Space Sciences, University of California, Los Angeles, CA 90095-1567 USA; 7 Dipartimento di Scienze Biologiche, Geologiche e Ambientali (BiGeA), Alma Mater Studiorum Università di Bologna, 40126 Bologna, Italy; 8 Dipartimento di Scienze della Terra, Università degli Studi di Torino, via Valperga Caluso 35, 10125 Torino, Italy

**Keywords:** Petrology, Geochemistry

## Abstract

Ocean sediments consist mainly of calcium carbonate and organic matter (phytoplankton debris). Once subducted, some carbon is removed from the slab and returns to the atmosphere as CO_2_ in arc magmas. Its isotopic signature is thought to reflect the bulk fraction of inorganic (carbonate) and organic (graphitic) carbon in the sedimentary source. Here we challenge this assumption by experimentally investigating model sediments composed of ^13^C-CaCO_3_ + ^12^C-graphite interacting with water at pressure, temperature and redox conditions of an average slab–mantle interface beneath arcs. We show that oxidative dissolution of graphite is the main process controlling the production of CO_2_, and its isotopic composition reflects the CO_2_/CaCO_3_ rather than the bulk graphite/CaCO_3_ (i.e., organic/inorganic carbon) fraction. We provide a mathematical model to relate the arc CO_2_ isotopic signature with the fluid–rock ratios and the redox state in force in its subarc source.

## Abstract (web summary)

The carbon isotopic signature of CO_2_ released from marine sediments subducted beneath volcanic arcs does not reflect their organic/inorganic fraction, but instead the fluid-rock ratios and the redox conditions in force at the top of the slab.

## Introduction

Modern open-ocean sediments are dominated by phytoplankton. Calcium carbonate, chiefly in sediments deposited above the calcite compensation depth, is essentially calcite forming the shells of organisms such as coccolithophores. It displays carbon isotopic ratios comparable to bicarbonate ions dissolved in seawater, characterized by δ^13^C ≈ 0‰ expressed as ^13^C/^12^C per mil difference normalized to the international Vienna-Pee Dee Belemnite (VPDB) standard. Conversely organic matter, which is essentially phytoplankton debris in the open seafloor, is depleted at ^13^C due to fractionation effects induced by photosynthesis, such that δ^13^C ≈ –20‰ VPDB^1^. Stable carbon isotopes and mass-balance calculations relying on simple mixing models have been used extensively to determine the relative contribution of organic matter and marine carbonates in sedimentary rocks^2^, in their metamorphic equivalents^3^ and in volcanic arc emissions^4^. However, the applicability of simple mixing models hinges on assumptions that may be overly simplistic, including that the sedimentary “end-member” compositions are spatially and temporally invariant, and that isotopic equilibrium is attained during metamorphism. The latter is particularly problematic because sedimentary organic carbon and coexisting carbonate may exhibit substantial isotopic exchange with increasing metamorphic grade, in particular at temperatures >650 °C during prograde graphitization^3,5–7^. In turn, fully crystalline graphite displays a sluggish rate of isotopic diffusion and may be unaffected by isotopic reset even in cases of intense metamorphism and fluid/rock interactions^8^.

Carbonates and graphitic carbon derived from organic matter, formerly assumed to be refractory to dissolution and devolatilization during subduction^9,10^, are now thought to show a non-negligible solubility in subduction fluids at least at certain *P*–*T*–*f*O_2_–pH conditions^11–18^. The interaction of subducted sediments with deep fluids^14,19^ produces dissolved carbon that is transferred from the slab to the overlying mantle wedge, prompting carbonation/metasomatism and/or partial melting^20–23^, and eventually returning to the surface via CO_2_ emitted by arc volcanoes^4^. The global arc average δ^13^C is −2.8 to −3.3 ‰^4^. This is heavier than the major carbon isotopic composition signature of the upper mantle (δ^13^C = −6.0 ± 2.5‰)^4^, but significantly lighter than sedimentary carbonates (δ^13^C ≈ 0‰^24^). As it is generally assumed that the carbon isotope signature of arc emissions reflects that of their source, relatively high δ^13^C values would point out assimilation of shallow “crustal” limestones, while low δ^13^C are usually attributed to subducted organic carbon^4^. However, processes of dissolution and of isotopic exchange involving organic and inorganic carbon beneath arcs are still not fully understood.

In this work, we investigate the carbon isotopic exchange occurring in a model system representative of open-ocean sediments containing calcium carbonate + organic matter subducted at subarc depths and interacting with aqueous fluids rising from the underlying dehydrating oceanic lithosphere^25^. We provide the quantitative chemical analysis of the volatile species and the measured carbon isotopic composition of CO_2_ produced by dissolution in water of graphite and of aragonite at *P* = 3 GPa, *T* = 700 °C and at redox-controlled conditions buffered to *f*H_2_ = FMQ (equivalent to *f*O_2_ expressed as ∆FMQ = + 0.61 log units). These conditions are selected on the basis of the predicted peak of CO_2_ produced by oxidative dissolution of graphite in subduction zones (Supplementary Fig. 1). Starting materials of synthetic labelled CaCO_3_ (99.4% ^13^C) and synthetic graphite (98.9% ^12^C) are used as analogues for natural “heavy” carbonate and “light” organic matter and to generate a maximum isotopic difference in experiments (close to pure ^13^C and ^12^C end-members). Control experiments are performed with oxalic acid di-hydrate (98.7% ^12^C) as the source of CO_2_ instead of graphite. The dissolution process and the effect of different CO_2_/aragonite ratios and of different run durations from 0.24 to 240 h are evaluated and compared with thermodynamic calculations that include consideration of aqueous solute speciation^26–28^ or consider only gas speciation in a conventional graphite-saturated COH fluid model^29,30^. Finally, a comparison with conventional isotopic models is provided. Experimental data allow the development of mathematical models extending the applicability of the results to a wide range of redox conditions and of fluid/rock ratios in order to predict the δ^13^C of CO_2_ released from the sedimentary slab and to envisage conditions required to meet the global average arc signature.

## Results and discussion

### CO_2_ evolved by the aqueous dissolution of aragonite-only, graphite-only and mixed aragonite + graphite

**Fig. 1:**
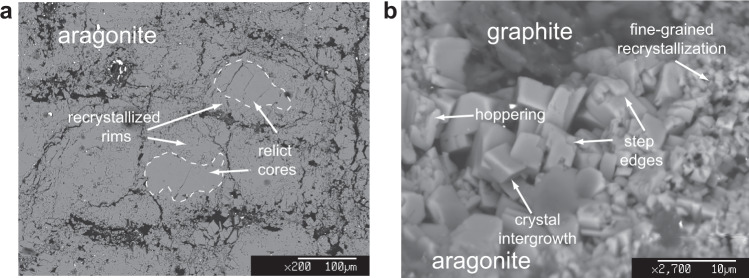
Electron microscope images showing microtextures of run products. **a** Recrystallized aragonite, showing rims of sub-micrometric crystals surrounding relict cores 50–100 µm in size. **b** Assemblage graphite + aragonite; aragonite crystals show dissolution/reprecipitation microtextures such as crystal size reduction, hoppering, step edges and euhedral crystal intergrowth.

In all experiments we observed that aragonite crystals in run products displayed textural evidence of dissolution-reprecipitation, including step edges, and fine-grained recrystallized rims around larger relict cores (Fig. 1). Conversely, we never observed texturally precipitation of newly formed graphite or graphite recrystallization, nor evidence for graphite isotopic variation compared to its starting composition.

**Fig. 2:**
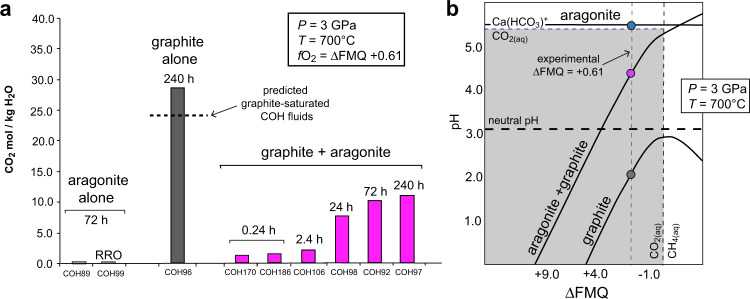
Experimental CO_2_ contents and thermodynamic modelling of fluids. **a** CO_2_ concentration (molality) in experimental aqueous fluids interacting with (i) aragonite-only (blue), (ii) graphite-only (grey) and (iii) graphite + aragonite (magenta). Typical analytical uncertainty is 1 mol%. Dashed line: CO_2_ content predicted by thermodynamic modelling of graphite-saturated COH fluids. Run duration (h) is shown at the top of each bar. RRO: run buffered by Re–ReO_2_ (ΔFMQ ≈ +2) instead of ferrosilite + magnetite + coesite; **b** *f*O_2_ vs. pH diagram at 3 GPa and 700 °C generated by thermodynamic modelling (Deep Earth Water model^26,28^) of aqueous fluids in equilibrium with aragonite-only (blue dot), graphite-only (grey dot) and aragonite + graphite (magenta dot). Black solid lines: saturation curves. Coloured dots: experimental conditions at *f*O_2_ buffered by ferrosilite + magnetite + coesite. Grey field: CO_2(aq)_ is the dominant carbon-bearing species; it is adjacent to Ca(HCO_3_)+-dominated field at higher pH and to CH_4(aq)_-dominated field at lower *f*O_2_ values. Neutral pH is shown for reference with a dashed line. Calculations are performed at 3 GPa, 700 °C and *f*H_2_ buffered by ferrosilite + magnetite + coesite + H_2_O (equivalent to log (*f*O_2_ /1 bar)= −13.36; ΔFMQ = +0.61).

In aragonite-only runs, despite the evidence of dissolution–precipitation microtextures, the absolute amount of CO_2_ evolved by the aqueous dissolution of aragonite-only is very low, close to the analytical detection limit, with *X*CO_2_ [=CO_2(aq)_/(H_2_O + CO_2(aq)_)_molar_] = 0.001 corresponding to 0.041 mol CO_2_/kg H_2_O (Fig. 2a; Supplementary Tables 1 and 2). We compared this result with thermodynamic modelling performed at the investigated *P*–*T*–*f*O_2_ conditions using the Deep Earth Water (DEW) model^26,28^ (Fig. 2b; Supplementary Table 3). Calculations indicate that fluids in equilibrium with pure aragonite should display a basic pH = 5.09 (neutral pH = 3.09 at 3 GPa and 700 °C) and *X*CO_2_ = 0.0002, which is even lower than the measured value. However, the model predicts that the dominant carbon-bearing dissolution product of aragonite at the investigated conditions is the calcium-bicarbonate ion Ca(HCO_3_)^+^, in agreement with previous studies^13,14^. According to the model, a substantial concentration of carbon is present in the form of ionic species. In particular, Ca(HCO_3_)^+^ accounts for the 74.3 mol% of the total carbon-bearing dissolved species, while CO_2(aq)_ only 7.3 mol% (Supplementary Table 3). The solubility of aragonite is therefore higher than that inferred on the basis of the measured CO_2_ only. As Ca(HCO_3_)^+^ is not measurable by QMS, we rely on the predicted Ca(HCO_3_)^+^ abundance to correct up the bulk aragonite solubility to 0.459 mol CO_2_/kg H_2_O, equivalent to 5.28 × 10^3^ ppm (=mg C/kg solution; 495 ppm of which deriving from measured CO_2(aq)_), which agrees well with previous estimates^14^. In aragonite-only runs, the evolved CO_2_ is independent of *f*O_2_, as demonstrated by the nearly identical *X*CO_2_ = 0.002 in the more oxidized (≈ΔFMQ + 2) control run buffered by Re–ReO_2_ (RRO in Fig. 2a; Supplementary Tables 1 and 2). On the contrary, the addition of ~0.5 molal to ~1 molal chlorine to lower pH in control experiments effectively boosts fluid CO_2_ concentrations to 2.17–2.46 mol CO_2_/kg H_2_O (corresponding to *X*CO_2_ = 0.038–0.042; Supplementary Tables 1 and 2).

Compared to runs with aragonite-only, the CO_2_ amount in fluids in experiments with graphite-only is significantly higher, with *X*CO_2_ = 0.339 after 240 h (Fig. 2a) corresponding to 28.5 mol CO_2_/kg H_2_O and a carbon concentration of 1.52 × 10^5^ ppm, two orders of magnitude higher than in fluids dissolving aragonite-only (Fig. 2a; Supplementary Tables 1 and 2). DEW model calculations show that fluids in equilibrium with pure graphite should display an acidic pH = 2.21 (Fig. 2b) and *X*CO_2_ = 0.305 (Supplementary Table 3), which is nearly identical to the value of *X*CO_2_ = 0.303 predicted by conventional modelling of graphite-saturated COH fluids^29,30^ (Fig. 2a; Supplementary Table 4) and close to the measured value of 0.339. In fluids in equilibrium with graphite-only, CO_2(aq)_ is predicted to account for 99.93% of the total dissolved carbon (Supplementary Table 3).

The CO_2_ amount measured in time-resolved runs containing mixed aragonite and graphite (Fig. 2a; Tables 1 and 2) ranges from *X*CO_2_ = 0.026 after 0.24 h to 0.166 after 240 h. Results suggest that chemical equilibrium is almost reached after 72 h. After 240 h, fluids contain 11.19 mol CO_2_/kg_water_ corresponding to 8.96 × 10^4^ ppm C, about one half that in fluids in equilibrium with graphite-only. This decline is not anticipated by either the DEW model calculations–which predict only a slightly lower *X*CO_2_ of 0.284 for fluids in equilibrium with graphite-only (Supplementary Table 3)—or by the graphite-saturated COH fluid model—which cannot account for the dissolution of aragonite because it does not consider components other than C, O and H. Our experimental result confirms that the estimation of *X*CO_2_ with available thermodynamic models is hampered in complex systems^15^. Nevertheless the DEW model is still useful to predict that fluids in equilibrium with both aragonite and graphite display a basic pH = 4.2 (Supplementary Table 3), which is higher than fluids in equilibrium with graphite-only but lower than fluids in equilibrium with aragonite-only, dominated by the dissolution product Ca(HCO_3_)^+^ (Fig. 2b). The consequence is that, as in fluids in equilibrium with graphite alone, CO_2(aq)_ is calculated to predominate in fluids in equilibrium with both graphite and aragonite (Fig. 2b), with CO_2(aq)_ accounting for 91.4% of the total carbon-bearing species, whereas Ca(HCO_3_)^+^ constitutes only 3.70% of the total carbon in solution (Supplementary Table 3).

### Carbon isotopic composition of graphite, aragonite and CO_2_

In experiments where water interacts with single minerals, the isotopic abundances of evolved CO_2_ are identical to that of the starting materials within analytical uncertainties. In particular, due to the analytical sensitivity of QMS, this is evident in runs characterized by higher CO_2_ production, i.e. ^13^C-aragonite + chlorine and ^12^C-graphite-only runs. In graphite-alone runs, the average CO_2_ isotopic abundance is 1.11% ^13^C after 240 h (Supplementary Table 1), indistinguishable from the ^13^C abundance of the starting graphite (1.09% ^13^C measured by IRMS; Supplementary Fig. 2) considering analytical uncertainties.

**Fig. 3:**
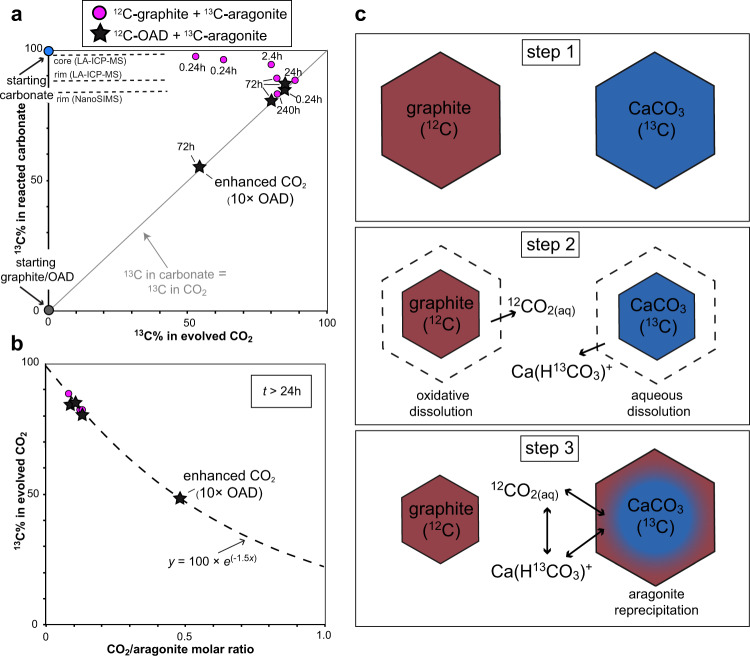
Isotopic composition of experimental fluids and solids. **a** Measured ^13^C abundance (%) in CO_2_ versus in bulk after-run aragonite. Grey reference line indicates identical ^13^C abundances in CO_2_ and in bulk aragonite. Starting ^13^C% of carbonate and graphite are indicated with blue and grey dots, respectively. Representative point analyses of aragonite cores and rims are shown with black dashed lines. Magenta dots: ^13^C-CaCO_3_ + graphite runs. Black stars: ^13^C-CaCO_3_ + oxalic acid di-hydrate (OAD; starting ^13^C% is coincident with starting graphite) runs. **b** CO_2_/^13^C-CaCO_3_ molar ratio plotted against ^13^C abundance (%) in CO_2_ of runs with duration >24 h. Dashed line represents the best fit of the data using an exponential equation. **c** Conceptual model for the carbon isotope exchange observed in experiments. At step 1, isotopically pure ^12^C-graphite (brown) and ^13^C-aragonite (blue) coexist in the same assemblage. As the interaction with water starts (step 2), graphite undergoes oxidative dissolution forming ^12^CO_2(aq)_, while aragonite dissolves forming Ca(H^13^CO_3_)^+^, as predicted by thermodynamic modelling. While graphite oxidative dissolution is self-limiting because the maximum amount of CO_2(aq)_ in the fluid is constrained by the redox state of the system, aragonite undergoes a continuous process of dissolution/precipitation (step 3). Therefore, the final isotopic composition of CO_2(aq)_, which is in dynamic equilibrium with Ca(HCO_3_)^+^ and thus with aragonite, becomes rapidly enriched in ^13^C ending with ^13^C abundances of >80% of the starting ^13^C-aragonite.

The isotopic signature of evolved CO_2_ in experiments with both ^13^C-aragonite and ^12^C-graphite (Fig. 3a; Supplementary Table 1) is relatively rich in ^12^C (^13^C = 52.9–62.8%) only in runs characterized by a very short duration of 0.24 h, while it becomes relatively homogeneous for *t* ≥ 24 h reaching ^13^C abundance = 82.2% after 240 h. Bulk measurements by means of total carbonate HCl dissolution allowed retrieval of the final average isotopic composition of after-run aragonite coexisting with CO_2_ (*y*-axis in Fig. 3a; Supplementary Table 1). Low ^13^C abundances in evolved CO_2_ are always coupled with high ^13^C in bulk aragonite, the latter tending to the starting ^13^C-CaCO_3_ value of 99.4% ^13^C (blue dot in Fig. 3a) in the shortest 0.24 h (^13^C = 96.1–97.2%) and 2.4 h (^13^C = 95.8%) runs (Fig. 3a; Supplementary Table 1). In longer *t* ≥ 24 h runs, ^13^C abundances of CO_2_ and of bulk carbonate converge (cf. black dashed line in Fig. 3a). After 240 h, the ^13^C abundance in aragonite is 82.9%, nearly identical to that of evolved CO_2_ (i.e. 82.2%).

Micro-analyses performed by means of NanoSIMS and LA-ICP-MS (dashed lines in Fig. 3a; Supplementary Tables 5 and 6) show that aragonite crystals are isotopically zoned, in particular in short runs, with recrystallized rims characterized by ^13^C abundances comparable to that of bulk aragonite and of CO_2_ in longer runs (e.g. ^13^C = 81.6(8)% in the 24 h run COH106; Supplementary Table 6), and relict cores showing high ^13^C abundance up to 98(1)% ^13^C (e.g. run COH97; Supplementary Table 6) approaching the starting composition of ^13^C-CaCO_3_. Conversely, the ^13^C abundance in after-run graphite indicates negligible isotopic re-equilibration (Supplementary Fig. 2; Supplementary Table 5, 6). NanoSIMS measurements range from 1.132(6)% to 1.195(3)% (Supplementary Fig. 2; Supplementary Table 5), concordant with LA-ICP-MS measurements ranging from ^13^C = 1.1(1)% to 1.5(2)% (Supplementary Fig. 2; Supplementary Table 6).

The ^12^C-rich composition of CO_2_ in the shortest runs and the lack of newly formed graphite suggest that in mixed aragonite–graphite runs, the early source of CO_2_ was graphite undergoing irreversible oxidation. To validate this hypothesis, we performed a series of control experiments at identical experimental conditions but without graphite, where the early source of ^12^CO_2_ is provided by oxalic acid di-hydrate (^13^C = 1.29%; OAD and black stars in Fig. 3a), which decomposes already at *T* ≈ 200 °C to a mixed H_2_O–CO_2_ fluid^31^. OAD has been added in the proper amount to keep the same CO_2_/^13^C-CaCO_3_ ratio ≈ 0.1 characterizing graphite + aragonite experiments (Fig. 3b; Supplementary Table 1). In addition, 10 times more OAD was added in a single run to increase the CO_2_/^13^C-CaCO_3_ ratio to a value of ~0.5 (“enhanced CO_2_” in Fig. 3a, b; Supplementary Table 1). In OAD + ^13^C-CaCO_3_ runs with CO_2_/^13^C-CaCO_3_ ratio ≈ 0.1, the isotopic composition of both CO_2_ and bulk carbonate shows no appreciable differences compared to aragonite + graphite runs, further supporting the hypothesis of graphite as the early source of CO_2_. In addition, the OAD + ^13^C-CaCO_3_ run with CO_2_/^13^C-CaCO_3_ ratio ≈ 0.5 makes evident that the ^13^C abundance of CO_2_ (and of coexisting aragonite) varies as a function of the CO_2_/^13^C-aragonite ratio (CAR), which can be described by the following exponential function (Fig. 3b):

1 $${}^{13}{{{{{\rm{C}}}}}}_{{{{{{{\rm{CO}}}}}}}_{2}} \% =100\times {e}^{\left(-1.5\times \!{{{{{\rm{CAR}}}}}}\right)}$$

which represents a mathematical model for the competing isotopic buffering in fluids where CO_2_ originated from the oxidative dissolution of ^12^C-graphite interacts with ^13^C-aragonite. Equation 1 shows that graphite-derived CO_2_ (and coexisting aragonite) are fully isotopically buffered by ^13^C-aragonite to ^13^C abundances ≈ 100% when CAR tends to zero. Conversely, by increasing CAR the contribution of ^12^C-graphite to the isotopic composition of CO_2_ becomes increasingly important, with ^13^C abundances tending to zero (i.e. fully buffered by graphite) at very high CO_2_/^13^C-aragonite ratios (e.g. ^13^C_CO2_ = 0.01 when CAR = 6).

### A conceptual model for carbon isotopic exchange among graphite, aragonite and CO_2_

In runs containing mixed ^13^C-aragonite and ^12^C-graphite, the isotopic composition of CO_2_ after 24 h is nearly coincident with that of the recrystallized aragonite, although it is produced mainly by the oxidative dissolution of graphite which remains isotopically unchanged. Our experimental results confirm that carbon isotope exchange between graphite and aragonite at 700 °C is sluggish, in agreement with previous findings suggesting that carbon diffusion in graphite is very slow at *T* < 1300 °C^ 32,33^. In the shortest 0.24 h and 2.4 h runs, despite the similar ^13^C abundances of CO_2_ and aragonite rims, the bulk carbonate ^13^C is markedly higher because of a number of unreacted ^13^C-rich cores persist, as shown by NanoSIMS and LA-ICP-MS analyses. In order to develop a conceptual model for the isotopic exchange among aragonite, graphite and CO_2_, we rely on observed microtextures, measurements and thermodynamic modelling results suggesting that during the run both graphite and aragonite undergo dissolution. However, ^12^C-graphite produces mainly ^12^CO_2_ by irreversible oxidation, which can be expressed by the following *f*O_2_-dependent reaction:

2 $$^{12}{{\rm{C}}}+{{{{{\rm{O}}}}}}_{2}= > ^{12}{{{{{\rm{CO}}}}}}_{2}$$

^13^C-aragonite dissolves initially forming mainly calcium-bicarbonate ions (Ca(H^13^CO_3_)^+^; Fig. 3c), according to the reactions involving the predominant species:

3 $${{{{{{\rm{Ca}}}}}}{}^{13}{{\rm{C}}}{{{{{\rm{O}}}}}}_{3}+{{{{{\rm{H}}}}}}_{2}{{{{{\rm{O}}}}}} < = > {{{{{\rm{Ca}}}}}}({{{{{\rm{H}}}}}}{}^{13}{{{{{\rm{CO}}}}}}_{3})^{+}+{{{{{\rm{OH}}}}}}}^{-}$$

when the fluid becomes saturated with respect to graphite, CO_2_ production by graphite oxidation stops and the unreacted graphite remains chemically and isotopically inert, while aragonite continuously dissolves and reprecipitates in dynamic equilibrium with the dissolved aqueous carbon species CO_2_ and Ca(HCO_3_)^+^, which in turn exchange carbon isotopes according to the following reaction:

4 $$^{12}{{{{{\rm{CO}}}}}}_{2} +{{{{{\rm{Ca}}}}}}({{{{{\rm{H}}}}}}{}^{13}{{{{{\rm{CO}}}}}}_{3})^{+} < = > {}^{13}{{{{{\rm{CO}}}}}}_{2}+{{{{{\rm{Ca}}}}}}({{{{{\rm{H}}}}}}{}^{12}{{{{{\rm{CO}}}}}}_{3})^{+}$$

therefore, because of the continuous dissolution/reprecipitation process controlled by Eq. 3, the carbonate is effective in buffering the isotopic signature of CO_2_ even after a very short time, despite the fact that CO_2_ is produced mainly by irreversible oxidation of graphite. Equation 1, however, predicts that aragonite buffering is possible only for relatively low CO_2_/aragonite ratios, ideally below 0.46 where the ^13^C abundances would be >50%. We will show below that Eq. 1 can be used to evaluate how the isotopic compositions of CO_2_ evolved from the sedimentary slab at subarc conditions can meet the global average arc signature according to those variables that control the CO_2_ release, i.e. the fluid/rock ratios and the redox conditions imposed by the environment.

### A mathematical model for δ^13^C of CO_2_ produced in the sedimentary slab and comparison with global average arcs

Thermodynamic considerations require that at graphite saturation and at fixed *P*–*T* conditions the amount of CO_2_ produced by oxidation of graphite in a pure, carbonate-free C–O–H system depends solely on: (i) redox conditions—the more oxidizing they are, the higher the *X*CO_2_ (=CO_2,aq_/(H_2_O+CO_2,aq_))^34^, and (ii) the amount (number of moles) of H_2_O interacting with graphite—the higher it is, the higher the amount of CO_2_ at fixed *X*CO_2_^30,35^. Therefore, at graphite saturation conditions and as long as graphite is present, the amount of CO_2_ produced by oxidation is not dependent on the amount of graphite in the system.

**Fig. 4:**
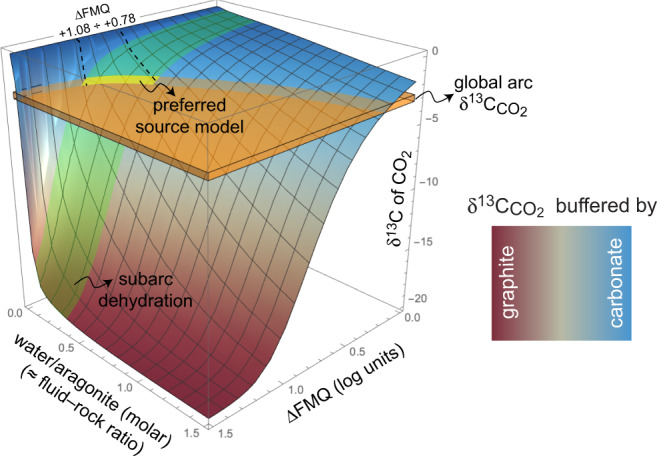
Mathematical model showing the predicted δ^13^C of CO_2_ as a function of both ∆FMQ and water–aragonite (≈ fluid–rock) molar ratio. Modelled CO_2_ is originated from oxidative dissolution of graphite and interacts with aragonite and aqueous fluids at 3 GPa and 700 °C. Brown surface color: δ^13^C buffered by graphite. Blue surface color: δ^13^C buffered by aragonite. Orange box: global average δ^13^C of arc CO_2_^4^; the intersection with the mathematical model shows that arc CO_2_ signatures are met for a wide range of ΔFMQ and fluid–rock ratios. Preferred model (yellow field) corresponds to the intersection among our mathematical model, the global average arc δ^13^C and typical fluid–rock ratios at slab-top conditions assuming percolating flux (green field; 0.05–0.33 molar = 0.01–0.06 mass). In this case, ΔFMQ ranging from +0.78 to +1.08 are derived for the source of carbon in the sedimentary slab at subarc conditions.

In graphite + aragonite + H_2_O systems, we demonstrated that the carbon contribution to fluids due to aragonite dissolution is negligible at subarc *P*–*T* conditions compared to graphite, so the amount of CO_2_ evolved still depends on (i) and (ii) above, with the additional consequence that the graphite/carbonate ratio is again not relevant. Moreover, the carbon content in these fluids is basically ascribable to their CO_2_ content, as Ca(HCO_3_)^+^ is limited to about 1%. However, we noticed a difference in the aragonite + graphite system compared to the graphite-alone (COH) system: the halving of measured *X*CO_2_ (=0.166) compared to graphite-saturated COH fluid models (=0.303; Fig. 2a). By analogy with other systems, we suggest that this is likely related to a modification of water activity^15^, in this case due to aragonite dissolution. Applying the experimentally derived correction factor of 0.548 (=0.166/0.303), we calculated for the aragonite + graphite system the *X*CO_2_ values predicted by conventional graphite-saturated fluid thermodynamic model^29,30^ at different redox conditions (ΔFMQ). The calculated *X*CO_2_ were used to calculate the absolute amount of CO_2_ produced by oxidation of graphite, obtained by fixing the absolute amount (moles) of H_2_O in the system. Assuming 1 mol aragonite in the system, fixed H_2_O values correspond also to water/aragonite molar ratios (WAR) and CO_2_ values to CO_2_/aragonite molar ratios. Using these last values and Eq. 1, we calculated the ^13^C abundance of CO_2_ produced by oxidation of ^12^C-graphite after interaction with ^13^C-aragonite. Because in our experimental model ^12^C-graphite and ^13^CaCO_3_ represent analogues of, respectively, ocean organic matter with δ^13^C ≈ −20 and marine carbonates with δ^13^C ≈ 0, we are now able to translate ^13^C abundances (ranging from 0 to 100%) to equivalent δ^13^C values (ranging from 0‰ to –20‰) using a conventional linear model:

5 $${\delta }^{13}{{{{{\rm{C}}}}}}\textperthousand =0.2{\times }^{13}{{{{{\rm{C}}}}}} \% {-}20$$

the calculated δ^13^C‰ of CO_2_ repeated for 10,000 different random combinations of the parameters ΔFMQ and WAR have been fitted with a three-variate parametric equation to provide, with an estimated average uncertainty of ±0.2‰, the following mathematical model (Fig. 4):

6 $${{{{{\rm{\delta }}}}}}{}^{13}{{{{{\rm{C}}}}}}_{{{{{{{\rm{CO}}}}}}}_{2}}{{\textperthousand }}=-20+20\frac{1}{1+{e}^{-f(\triangle {{{{{\rm{FMQ}}}}}},{{{{{\rm{WAR}}}}}})}}$$

where the function *f*(ΔFMQ, WAR) is a third-order polynomial found to minimize residuals (parameters and associated uncertainties available in Supplementary Table 7). The global arc δ^13^C ranging from –2.8 to –3.3 ‰^4^ (orange box in Fig. 4) intersects the surface of Eq. 6 providing a continuous layer that can be averaged and expressed with the following equation:

7 $$\Delta {{{{{\rm{FMQ}}}}}}=\frac{2}{1+{e}^{(A+B\times {{{{{\rm{WAR}}}}}}+C{\times {{{{{\rm{WAR}}}}}}}^{2}+D\times {{{{{{\rm{WAR}}}}}}}^{3})}}$$

equation 7, where *A* = –0.314(9), *B* = 2.75(5), *C* = –1.46(6) and *D* = 0.37(2), represents the locus of points of all the possible combinations of ΔFMQ and water/aragonite ratio that result in the isotopic composition of sediment-derived CO_2_ satisfying the global average arc values.

### Comparison with conventional mass-balance/equilibrium fractionation models

The observed carbon isotopic exchange among CO_2_, graphite and aragonite is potential of interest for subducted sediments buried in a wide range of basins; the only limiting factor is the coexistence of calcium carbonate with elemental carbon (formerly organic matter) in whatever proportion and the presence of an aqueous fluid. Our experiments at 3 GPa and 700 °C show that CO_2_ produced by oxidative dissolution of ^12^C-graphite (^13^C = 1.1%; equivalent δ^13^C = –19.8, cf. Eq. 5 and Supplementary Table 1) becomes rapidly enriched in ^13^C ending after 240 h with ^13^C = 82.2% (equivalent δ^13^C = –3.56) due to its isotopic exchange with ^13^C-aragonite (^13^C = 99.6%; equivalent δ^13^C = –0.08), despite the overwhelming abundance of graphite in the starting materials (graphite/aragonite_(molar)_ = 5.6; see below).

**Fig. 5:**
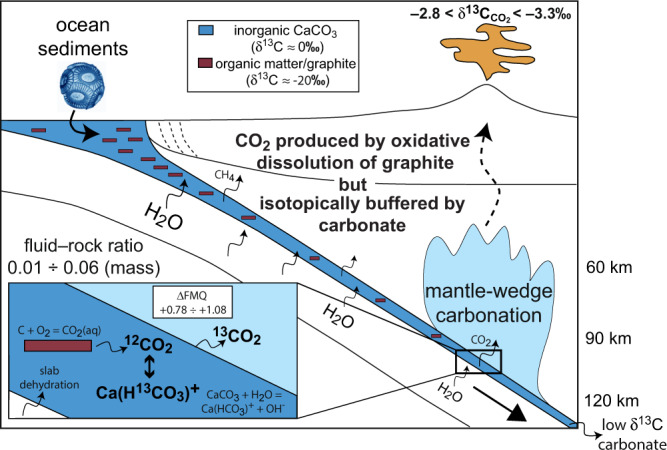
Carbon isotope exchange at the subarc slab-mantle interface. Organic (brown) and inorganic (dark blue) carbon occurring in open-ocean sediments enters the subduction channel, where it interacts with aqueous fluids produced by dehydration of the down-going oceanic lithosphere. While calcium carbonate dissolution increases almost linearly with depth^14^, resulting in fluids containing ~5000 ppm C mainly as Ca(HCO_3_)^+^ at subarc depth, the oxidative dissolution of graphite increases exponentially and reaches its maximum at subarc conditions, where fluids coexisting with graphite contain ~140,000 ppm C mainly under the form of CO_2(aq)_. With the simultaneous presence of aragonite and graphite, experiments show that the carbon concentration in aqueous fluids is halved, but still controlled by oxidation of graphite to CO_2_. Nevertheless, as aragonite is very prone to dissolution-reprecipitation processes, it shows an outstanding capacity to buffer the isotopic signature of graphite-derived CO_2_ to carbonate-like values characterizing the global arc CO_2_ (orange field). In order to exert its buffering capacity, graphite-derived CO_2_ must be low with respect to carbonate, which is the case for low fluid–rock ratios coupled with oxidizing conditions (preferred model) or for high fluid–rock ratios coupled with relatively reducing conditions. We suggest that, as a mobile but reactive fluid-phase, CO_2_ is particularly effective in metasomatizing the supra-subduction mantle (light blue field), eventually imposing its isotopic signature to the deep source of arc magmatism.

Equivalent δ^13^C values can be compared with conventional models combining mass-balance calculations with isotope equilibrium fractionation (see Methods). Let’s assume a mixture similar to what investigated experimentally: aragonite (δ^13^C = 0‰, by analogy with a marine carbonate source) + graphite (δ^13^C = –20‰; by analogy with a marine organic matter source), equilibrating at 700 °C (pressure effect ignored). Assuming complete solid–solid isotopic exchange and neglecting devolatilization, the final isotopic composition would be a function of the molar ratio between graphite and aragonite, for instance: 1) δ^13^C_graphite_ = –5.29‰; δ^13^C_aragonite_ = –0.14‰ for graphite/aragonite = 0.01; 2) δ^13^C_graphite_ = –12.57‰; δ^13^C_aragonite_ = –7.43‰ for graphite/aragonite = 1.0; 3) δ^13^C_graphite_ = –17.77‰; δ^13^C_aragonite_ = –12.63‰ for graphite/aragonite = 5.6. With the latter abundance value, comparable with the experimental setup, calculations show that the isotopic composition of all the phases in equilibrium would vary slightly as a function of the amount of CO_2_ in the system produced by oxidation of graphite (Supplementary Fig. 3a), with δ^13^C_CO2_ = –10.17‰ for CO_2_/aragonite = 0 and δ^13^C_CO2_ = –11.33‰ for CO_2_/aragonite = 1. This conventional model is therefore not adequate to reproduce our experimental system, characterized by CO_2_/aragonite ≈ 0.1, where both aragonite and CO_2_ converge to much heavier compositions. However, the fit between the isotopic model and the experimental trend improves significantly (Supplementary Fig. 3a) if we assume (1) graphite as chemically reactive but isotopically inert phase and (2) starting CO_2_ displaying δ^13^C = –20‰, i.e. the same value of the starting graphite. These assumptions, deduced on the basis of our experimental findings, underline again the need for experimental constraints on deep isotopic exchange processes involving fluids. For instance, an implication of our study could throw some light on the hard debate on the genesis of diamonds characterized by light, organic matter-like isotopic compositions^19^. In fact, even if we predict that, after having interacted at subarc conditions with graphite-saturated fluids, sedimentary carbonates will be only slightly depleted in ^13^C, showing the same δ^13^C −2.8 to −3.3‰ characterizing CO_2_, they could become locally lighter (cf. brown in Fig. 4) in case of highly oxidized conditions^36^ or very high water/carbonate (≈fluid/rock) ratios^37^. Because of their stability at high-pressure conditions^38^, these carbonates could be subducted further, eventually making available a ^12^C-enriched source of carbon, which comes from carbonate and not organic matter, in the diamond stability field^39^ (Fig. 5).

### The link between sedimentary slab fluids and arc emissions

As an aqueous fluid-phase species, CO_2_ originated from subducted sediments close to the slab-mantle interface is particularly effective in metasomatizing the subarc supra-subduction mantle, prompting carbonation reactions^20,21,23,40^ that may facilitate diapiric upwelling and melting processes^20,41^. Therefore, there is good reason to believe that sediment-derived CO_2_ can impose its isotopic signature on the subarc mantle, the source of arc magmatism (Fig. 5). The interaction of subducted ocean carbonates and organic matter with fluids coming from the dehydration of the down-going slab^25,42^ is a continuous process starting early in the forearc region^9,43^ (Fig. 5; Supplementary Fig. 1). However, our study indicates that the production of CO_2_ is strictly connected to the stability of graphite in aqueous fluids. At forearc conditions graphite is poorly soluble by oxidative dissolution, even considering the increased dissolution susceptibility for disordered graphitic carbon, in the absence of intense flushing by aqueous fluids (i.e. channelized flow)^16,35^. Aqueous fluids reach their maximum CO_2_ contents at subarc conditions (Fig. 5; Supplementary Fig. 1), which are the conditions investigated in our study^16^. Beneath arcs, the interaction between aqueous fluids coming from the dehydrating oceanic slab and percolating into the overlying carbonate metasediments will thus result in a large amount of CO_2_ leached out from the slab, as long as graphite is not completely removed by oxidative dissolution, which depends chiefly on the time-integrated fluid flux for given redox state^18^. Although channelized fluid flow can potentially dissolve the entire amount of organic carbon at blueschist-to-eclogite conditions, the geological record suggests the complete removal is difficult even for very high fluxes and often limited to the more soluble disordered forms of organic carbon^18^. Fully crystalline graphite, which is characterized by sluggish rates of isotopic diffusion^3^, is thus expected to persist at subarc depths. We demonstrated experimentally that the organic carbon content is not a relevant variable in controlling the carbon isotopic signature of CO_2_ produced by dissolution processes, which only depends on redox conditions and fluid-rock ratios. Therefore, δ^13^C of gaseous arc emissions is not a simple linear combination of the amount of buried organic and inorganic carbon^4^. One major implication is that fluctuations in δ^13^C_CO2_ do not necessarily reflect variations in the source input, for instance, due to shallow crustal carbonate assimilation^44,45^ or to the burial of anoxic sediments dominated by organic matter^19^. It could reflect instead local variations in fluid-rock ratios (i.e. channelized vs. percolating flux) or in the redox state (i.e. oxidizing vs reducing conditions). Equation 7 can be used to constrain the average redox state (expressed as ΔFMQ) in the sedimentary carbon source of the slab that meets the global arc δ^13^C_CO2_. ΔFMQ values ranging from +0.78 to +1.08 are predicted assuming a fluid–rock mass ratio (≈WAR) of 0.01–0.06 (=0.05–0.33 molar), which has been suggested for infiltrating, non-channelized fluids produced by dehydration reactions in eclogites^46,47^. These oxygen fugacity values are in agreement with estimations of island-arc-basalt (IAB) sources^48,49^ and supra-subduction mantle peridotites equilibrated at subarc depths^50^. It remains an open question how these oxygen fugacity values correlate with the amount of oxygen^36^ transferred from the slab to the IAB source, with consequences for the redox budget of subarc mantle^51^.

## Method

### Experimental approach and characterization of the solid- and fluid phases

In this study, we employed as starting materials: (i) labelled Ca^13^CO_3_ (calcite; forming aragonite at run conditions) (Sigma–Aldrich); (ii) synthetic graphite (Sigma–Aldrich), highly ordered as suggested by Raman spectroscopy^16^; (iii) oxalic acid di-hydrate (Sigma); (iv) MilliQ water, boiled while flushed with N_2_ to remove dissolved atmospheric CO_2_. Experiments were buffered using the double-capsule technique^52^ (Supplementary Fig. 4) with an inner H_2_-permeable Au_60_Pd_40_ capsule, containing the starting materials with the addition of water (~20 wt%), and an outer Au capsule filled with the buffering assemblage fayalite + magnetite + quartz + H_2_O (FMQ; forming ferrosilite + magnetite + coesite at run conditions, verified by electron microscopy, electron microprobe analysis and Raman spectroscopy; Supplementary Fig. 5). The buffer in the outer capsule constrains directly the *f*H_2_ in the inner capsule. The redox conditions (*f*O_2_) in the inner capsules are constrained indirectly by the buffer, whose ∆FMQ is +0.76 log units in the outer capsule, to slightly lower *f*O_2_ values (∆FMQ = +0.61 log units; see “Thermodynamic modelling” and Supplementary Table 4).

Experiments were performed at 3 GPa and 700 °C using an end-loaded piston-cylinder apparatus. Temperatures were measured with K-type thermocouples and considered accurate to ±5 °C. Pressure calibration is based on the quartz/coesite transition^53^ and accurate to ±0.01 GPa. Runs with CaCO_3_ were first pressurized at 3 GPa for 2 h, to promote carbonate crystal growth by cold sintering^54^ in order to increase the grain size of the synthetic micrometric CaCO_3_ power. Samples are then heated to 700 °C with a ramp of 100 °C/min. Run durations were from 2.4 to 240 h. Quench is obtained by cutting off the power supply, resulting in a temperature decline of >40 °C per second. CO_2_, water and other volatiles in the inner capsules were measured quantitatively by quadrupole mass spectrometry (QMS) using the capsule-piercing technique^31^. The typical analytical uncertainty is 1 mol% for CO_2_ and H_2_O. Solid phases were checked by scanning electron microscopy, electron microprobe analyses and micro-Raman spectroscopy.

### Isotopic analysis of CO_2_

The quantitative analysis and the determination of the ^13^C/^12^C ratio of CO_2_ have been performed simultaneously by means of QMS^31^, monitoring the *m*/*z* channel 45 (^13^CO_2_) in addition to channels considered for routine analysis. The calibration curve linking the ^13^C/^12^C ratio of CO_2_ and the ratio of the integrated peaks of channels 45 (^13^CO_2_) and 44 (^12^CO_2_) (44/45 ratio) has been derived by measuring 3 different mixtures with known ^13^C/^12^C ratio prepared to start from (i) regular oxalic acid di-hydrate (OAD; Sigma–Aldrich), used also for OAD + Ca^13^CO_3_ experiments and (ii) isotopically nearly pure ^13^C oxalic acid di-hydrate (Sigma–Aldrich), thermally decomposed to CO_2_ at 250 °C^31^ (Supplementary Table 8). Analyses performed by QMS are affected by a very small mass-bias effect. Actually, the regression line provided a very low correction factor of 1.03 for the integrated peak of channel 45.

### Isotopic analysis of solids: NanoSIMS

^13^C/^12^C ratios of carbonate and graphite grains in the experimental sample were determined by nanoscale secondary ion mass spectrometry (NanoSIMS). Measurements were conducted on the Cameca NanoSIMS 50 installed at Muséum National d’Histoire Naturelle of Paris. The sample capsule was included in pure indium and gold-coated (20 nm thick). Secondary ions of ^12^C^12^C^−^ and ^13^C^12^C^−^ were collected in multicollection mode to obtain ^13^C/^12^C ratios after calibrating with natural abundance graphite and calcite samples. ^16^O^−^ secondary ions were used to locate graphite versus carbonate grains. Mass resolving power was set at a minimum 10,000, enough to resolve interferences on measured secondary ions. Before each analysis, a 5 × 5 µm^2^ surface area was initially pre-sputtered for 60 s with a 500 pA primary Cs^+^ rastering beam, in order to remove the gold coating and reach a sputtering steady-state^55^. For analyses of graphite grains, the primary beam was set to 1 pA and is was scanned over a surface area of 5 × 5 µm^2^. Nevertheless, to avoid surface contamination, only ions from the inner 2.6 × 2.6 µm^2^ regions were collected with the “beam blanking mode”. Each analysis consisted of a stack of 100 cycles, with a duration of 2.048 s each. Similar settings were used to carbonate grains, except that the primary beam was set to 9 pA and each cycle was 8.192 s long. ^13^C/^12^C of carbonate grains were also investigated using ^12^C^−^ and ^13^C^−^ secondary ions (mass resolving power set to 9000), showing no significant deviation from the measurements using C_2_^−^ secondary ions. Due to large isotopic variations observed in these samples, instrumental mass fractionation (of a few per mil) was neglected.

### Isotopic analysis of solids: LA-ICP-MS

^13^C/^12^C ratios of carbonate and graphite grains in the experimental sample were also determined by LA-ICP-MS. Measurements were carried out over a New Wave UP 266 laser ablation system coupled with a Thermo Fisher ICAP-Q ICP-MS (University of Insubria). In order to find the best compromise between ablated surface and sensitivity, ablation conditions were optimized over a natural abundance calcite sample with a known ^12^C/^13^C ratio (see Section “Isotopic analysis of solids: Bulk analyses”). As a result, for each spot determination, 20 shots within 1 s were performed, using a 30 μm diameter circular spot size and adjusting the laser power to obtain a fluence value of around 17 J/cm^2^. Helium was used as the carrier gas (0.85 L/min). Under these conditions the hole depth (subsequently evaluated by scanning electron microscopy) is, on average, about 20 μm. Analytical accuracies and uncertainties have been evaluated by measuring the following internal standards: (i) natural abundance calcite, characterized isotopically by GasBench isotope ratio mass spectometer (IRMS) in two different laboratories (Florence and Milan), displaying an average ^13^C abundance of 1.1208(2) %; (ii) synthetic labelled CaCO_3_ produced by precipitation from a Na_2_^13^CO_3_ + Na_2_^12^CO_3_ solution treated with CaCl_2_, with ^13^C abundance of 43.8% measured by HCl dissolution followed by QMS analysis of evolved CO_2_ (see “Bulk analyses” below). LA-ICP-MS measurements on these standards have been proven to be accurate to ~2% for natural calcite (standard deviation 5.8%) and ~1% for synthetic ^13^C–^12^C calcite (standard deviation 0.7%) (Supplementary Table 9). Analyses of graphite are affected by a higher standard deviation of about 12%, due to decreased ablation efficiency.

### Isotopic analysis of solids: Bulk analyses

Bulk analyses of the carbonates contained in the experimental capsules have been carried out by HCl decomposition followed by QMS analysis of evolved CO_2_ (see “Isotopic analysis of CO_2_”). To perform this analysis sample capsules coming from LA-ICP-MS determination were placed in a U-shaped glass tube located upstream with respect to the QMS analyser. After 5 outgassing cycles with Ar, 1 mL of HCl (4.5 M) was introduced (from an additional port) in the bottom of the U-tube, where the capsule is located. The tube was then closed (by means of a bypass valve of the gas manifold) for 30 min in order to allow the complete decomposition of all carbonates: after this reaction time the tube was then placed in line with the QMS analyser to determine the composition of evolved CO_2_. The analysis of the natural calcite standard provided a ^13^C abundance of 1.13%, which is accurate to 0.8% of the certified value. In addition, this method allowed to retrieve the ^13^C abundance of the synthesized ^13^C–^12^C calcite (43.8%) and of Ca^13^CO_3_ used as starting material (99.4%) (Supplementary Table 10).

The isotopic characterization of the graphite used as starting material has been performed using EA-IRMS analysis, which yielded a value of 1.0948(2)% ^13^C.

### Thermodynamic modelling of fluid composition

To retrieve the redox conditions in the double-capsule system, oxygen and hydrogen fugacities have been calculated by conventional thermodynamic modelling (Supplementary Table 4). In the outer capsule, containing the buffering assemblage ferrrosilite + magnetite + coesite + H_2_O, log (*f*O_2_/1 bar)= –13.21 and log (*f*H_2_/1 bar) = 2.195 have been calculated at *P–T* conditions of 3 GPa and 700 °C using the Perple_X package^30^, considering the thermodynamic dataset of Holland and Powell^56^ revised by the authors in 2004 (hp04ver.dat) and the equation of state “H–O HSMRK/MRK hybrid” of the routine “fluids”. Then, *X*CO_2_ [=CO_2_/(H_2_O+CO_2_)_molar_] = 0.303 for graphite-saturated fluids has been calculated by fixing log (*f*H_2_/1 bar)= 2.195, which is homogeneous in the inner and the outer capsule, using the Perple_X equation of state of Connolly and Cesare^29^ (cf. refs. ^15,16^ for other details). This *X*CO_2_ corresponds to an inner-capsule log (*f*O_2_/1 bar)= –13.36 (ΔFMQ = +0.61 log units), which reflects the redox conditions occurring in the runs bearing graphite. Log (*f*H_2_/1 bar)= 2.195 has also been used to calculate the fluid speciation and the pH in our experimental systems, using the Deep Earth Water (DEW) model^26,27^ (Supplementary Table 3).

### Conventional isotopic modelling

The global δ^13^C of the investigated system can be expressed with the following mass balance:

8 $${\delta }^{13}{C}_{{{{{{\mathrm{global}}}}}}}={m}_{{{{{{\mathrm{graphite}}}}}}}\times {\delta }^{13}{C}_{{{{{{\mathrm{graphite}}}}}}\_i}+{m}_{{{{{{\mathrm{aragonite}}}}}}}\times {\delta }^{13}{C}_{{{{{{\mathrm{aragonite}}}}}}\_i},$$

where *m* represents the molar fractions, and δ^13^C_graphite_i_ and δ^13^C_aragonite_i_ the initial composition of graphite and aragonite, respectively, –20‰ and 0‰ in our isotopic model.

Assuming that CO_2_ is produced only through oxidation of graphite (graphite_ox), and that equilibrium conditions occur between CO_2_ and graphite, the isotopic composition of graphite_ox is:

9 $${\delta }^{13}{C}_{{{{\mathrm{graphite}}}}\_ox}=(1-f)\times ({\delta }^{13}{C}_{{{{\mathrm{graphite}}}}\_i}{-}{\Delta }_{{{{\mathrm{CO}}}_{2}}{-}{{{\mathrm{graphite}}}}})+f\times {\delta }^{13}{C}_{{{{\mathrm{graphite}}}}\_i}$$

where $\Delta_{{\mathrm {CO}}_{2}}$–graphite is the CO_2_–graphite equilibrium fractionation factor at 700 °C^57^ and *f* is the molar fraction of oxidated graphite, i.e. (*m*_graphite_ − *m*_CO2_)/*m*_graphite_, ranging from 1 (no CO_2_ produced) to 0 (graphite fully oxidated to CO_2_).

The isotopic composition of CO_2_ produced by graphite oxidation is:

10 $${\delta }^{13}{C}_{{{{{{\mathrm{CO}}}}}}_{2}}={\delta }^{13}{C}_{{{{{{\mathrm{graphite}}}}}}\_{ox}}+{\Delta }_{{{{{{\mathrm{CO}}}}}}_{2}{-}{{{{{\mathrm{graphite}}}}}}},$$

so that the lobal δ^13^C including CO_2_ (δ^13^C_global_ox_), which in a closed system is numerically identical to δ^13^C_global_, can be expressed as:

11 $${\delta }^{13}{C}_{{{{{{\mathrm{global}}}}}}\_ox} = \,	({m}_{{{{{{\mathrm{graphite}}}}}}}{-}{m}_{{{{{{\mathrm{CO}}}}}}_{2}})\times {\delta }^{13}{C}_{{{{{{\mathrm{graphite}}}}}}\_ox}+{m}_{{{{{{\mathrm{CO2}}}}}}}\times {\delta }^{13}{C}_{{{{{{\mathrm{CO}}}}}}_{2}}\\ 	+ \,{m}_{{{{{{\mathrm{aragonite}}}}}}}\times {\delta }^{13}{C}_{{{{{{\mathrm{aragonite}}}}}}\_i},$$

Assuming isotopic equilibrium between CO_2_, graphite_ox and aragonite (Supplementary Fig. 3a), the final compositions are:

12 $${\delta }^{13}{C}_{{{{\mathrm{aragonite}}}}\_f}\,=\,	{\delta }^{13}{C}_{{{{\mathrm{global}}}}\_ox}{-}({m}_{{{{\mathrm{graphite}}}}}\left.{-}{m}_{{{{\mathrm{CO}}}}_{2}}\right)\times {-}{\Delta }_{{{{\mathrm{aragonite}}}}{-}{{{\mathrm{graphite}}}}})\\ 	{-}({m}_{{{{\mathrm{CO2}}}}}\times {-}{\Delta }_{{{{\mathrm{aragonite}}}}{-}{{{\mathrm{CO}}}_{2}}});$$

13 $${\delta }^{13}{C}_{{{{{{\mathrm{graphite}}}}}}\_f}={\delta }^{13}{C}_{{{{{{\mathrm{aragonite}}}}}}\_f}{-}{\Delta }_{{{{{{\mathrm{aragonite}}}}}}{-}{{{{{\mathrm{graphite}}}}}}};$$

14 $${\delta }^{13}{C}_{{{{{{\mathrm{CO}}}}}}_{2}\_{f}}={\delta }^{13}{C}_{{{{{{\mathrm{aragonite}}}}}}\_f}{-}{\Delta }_{{{{{{\mathrm{aragonite}}}}}}{-}{{{{{\mathrm{CO}}}}}}_{2}},$$

In calculations considering graphite as isotopically inert (Supplementary Fig. 3b), we assume that CO_2_ is produced by the oxidation of graphite, but that CO_2_ does not equilibrate with graphite getting oxidized.

Therefore, only CO_2_ and aragonite are freely exchanging isotopes. In this case, *m*_graphite_ becomes 0 and δ^13^C_CO2_ displays the constant value of –20‰. Therefore, the following equations were used instead:

15 $${\delta }^{13}{C}_{global}={m}_{CO2}\times 20\textperthousand +{m}_{Aragonite}\times {\delta }^{13}{C}_{{aragonite}{\_}{i}};$$

16 $${\delta }^{13}{C}_{{{{{{\mathrm{aragonite}}}}}}{\_}{f}}={\delta }^{13}{C}_{{{{{{\mathrm{global}}}}}}}{-}({m}_{{{{{{\mathrm{CO2}}}}}}}\times {\Delta }_{{{{{{\mathrm{aragonite}}}}}}{-}{{{{{\mathrm{CO}}}}}_{2}}});$$

17 $${{\delta }^{13}} {C}_{{{{{{{\mathrm{CO}}}}}}_{2}}\_f}={\delta }^{13}{C}_{{{{{{{{\mathrm{aragonite}}}}}}}}\_f}{-}{\Delta }_{{{{{{{{\mathrm{aragonite}}}}}}}}{-}{{{{{{{{\mathrm{CO}}}}}_{2}}}}}}.$$

## Supplementary information

Supplementary Information

Peer Review File

## Data Availability

The authors declare that the data supporting the findings of this study are available within the article.

## Code availability

The authors declare that the codes developed for this study are available within the article.
