# Supplementary material for: Subducted organic matter buffered by marine carbonate rules the carbon isotopic signature of arc emissions
Source: Nat Commun. 2022 May 25;13:2909. doi: 10.1038/s41467-022-30421-5 (PMC9132964; doi:10.1038/s41467-022-30421-5)
Supplement: Supplementary file 1 — Supplementary Information [file 41467_2022_30421_MOESM1_ESM.pdf]

Supplementary Figure 1

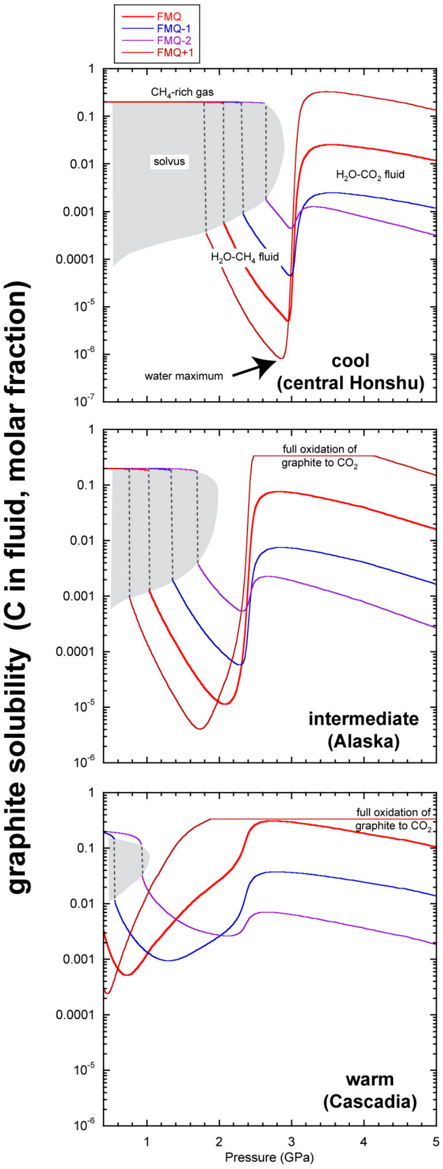

Graphite solubility (molar fraction C) in aqueous fluids buffered at different ΔFMQ conditions. Calculations are performed by conventional thermodynamic modelling of graphite-saturated COH fluids(*1*) for model cool, intermediate and warm subduction regimes. Regardless of geothermal gradient, the carbon content initially decreases with depth to a minimum, then increases over a narrow depth range and remains nearly constant to greater depth. The solubility pattern tracks a transition from CH_4 dominated fluids at shallow depths, and CO_2 dominated fluids at greater depth; the minima occur where the bulk XO = 1/3 (H_2O stoichiometry; "water maximum" conditions). Shallow fluid compositions intersect the H_2O–CH_4 miscibility gap.

Supplementary Figure 2

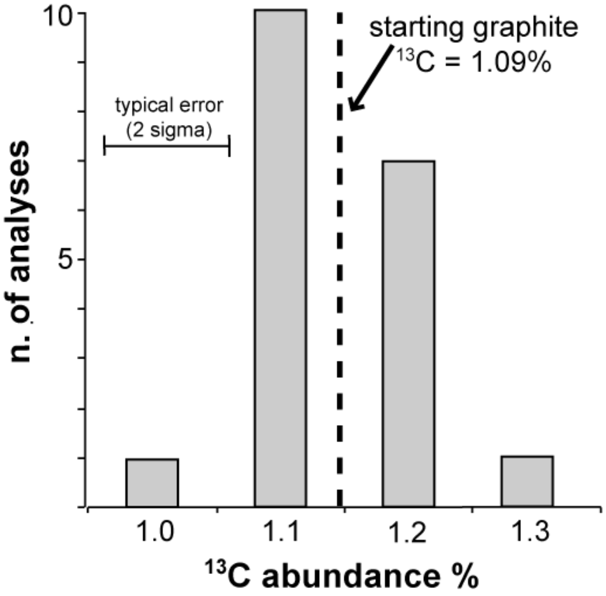

Histogram of the post-quench isotopic composition of graphite. Analyses are performed by means of NanoSIMS and LA-ICP-MS. Dashed line: isotopic composition of the starting graphite (EA-IRMS). Error bar represents typical uncertainty of LA-ICP-MS analyses.

Supplementary Figure 3

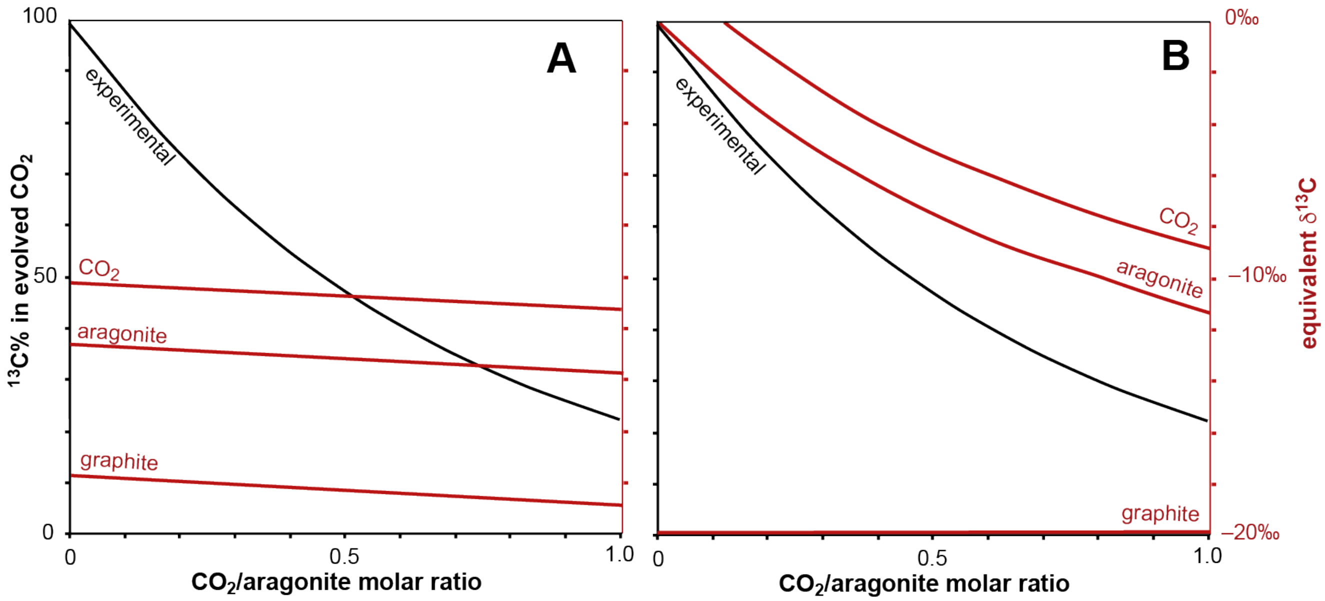

Comparison of the experimental data fit (solid black lines) with conventional isotopic modelling (solid red lines). Modelling is performed assuming $T = 700$°C, room pressure and that $\delta^{13}C = 0$‰ and –20‰ are equivalent to $^{13}C = 100\%$ and 0%, respectively. $CO_2/CaCO_3$ molar ratio is displayed in the x-axis. (A) In the conventional isotopic model, CO_2, aragonite and graphite can all freely exchange isotopes and reach mass-balance equilibrium. The experimental conditions are replicated with 30 µmol aragonite and progressive oxidation of 166.5 µmol of graphite to CO_2 from 0 µmol ($x = 0$) to 30 µmol ($\boldsymbol{x} = 1$); (B) graphite is treated as a chemically reactive phase, oxidizing to CO_2, but as an isotopically inert phase; in this case only aragonite and CO_2 can freely exchange isotopes to reach mass-balance equilibrium.

Supplementary Figure 4

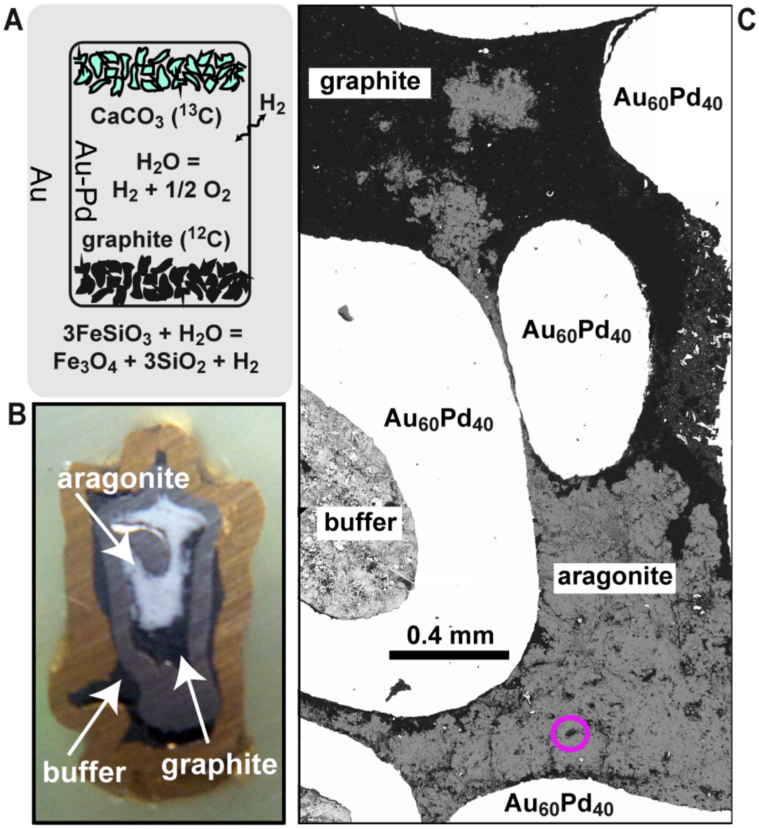

Details of the experimental design. (A) Outline of the typical experimental capsule; in the inner Au–Pd capsule, permeable to H_2, synthetic graphite and labelled $Ca^{13}CO_3$ interact with excess ultrapure water (~20 wt%). In the outer Au capsule, the buffering assemblage ferrosilite + magnetite + coesite constrains the hydrogen fugacity. The expected fO_2 in the inner capsule is ΔFMQ = –0.16. (B) Example of post-quench double capsule (sample COH98) containing the assemblage graphite + aragonite (checked by micro-Raman spectroscopy). Outer gold capsule diameter: 4.5 mm; natural light photograph. (c) Back-scattered electron image of the inner Au–Pd capsule of sample COH92 after NanoSIMS analysis. Size of a typical pit in aragonite is shown within the magenta circle.

Supplementary Figure 5

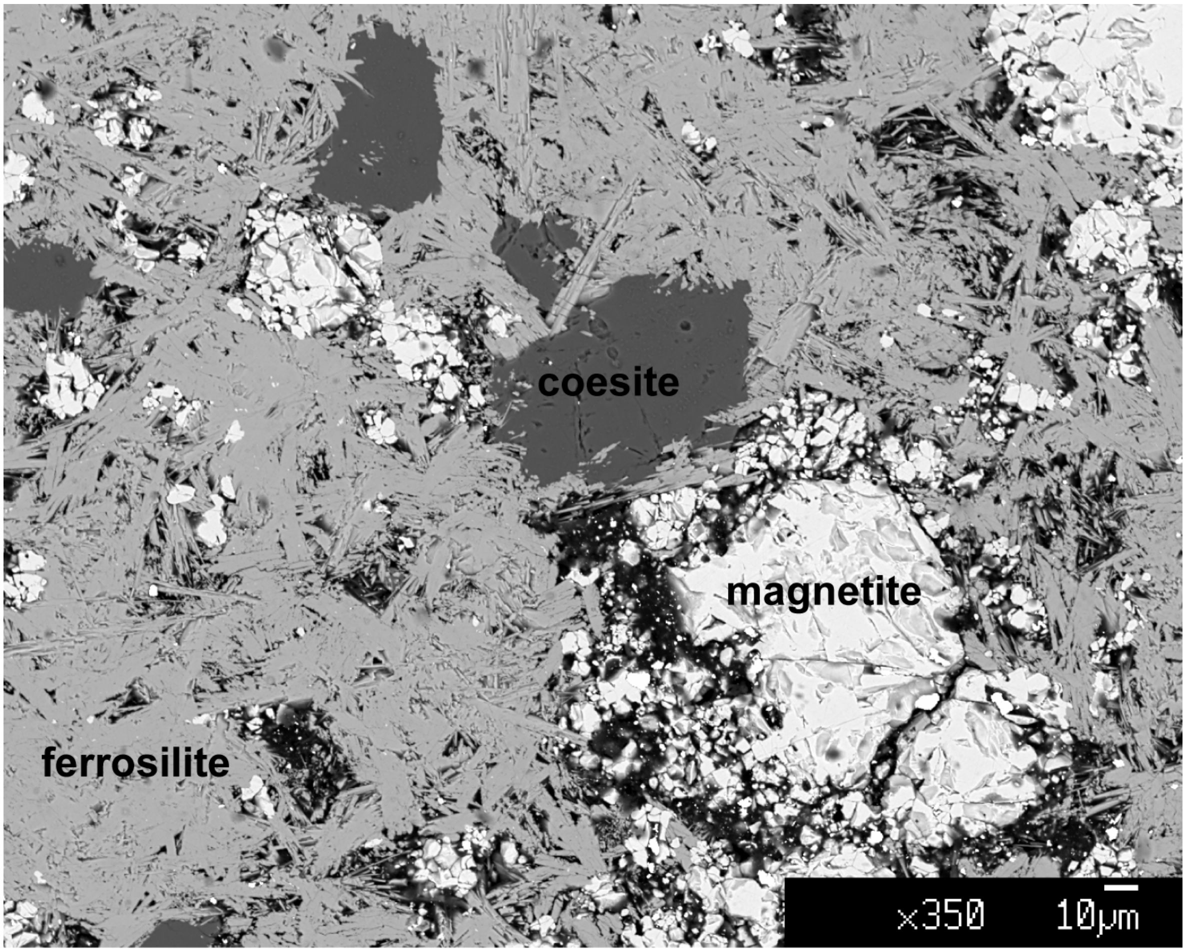

Back-scattered electron image of the buffering assemblage in the outer capsule. The occurrence of ferrosilite + magnetite + coesite is confirmed by electron-microprobe analysis and micro-Raman spectroscopy.

Supplementary Table 1. Run table of the experiments performed at 3 GPa–700°C and analyses of volatiles and of bulk after-run aragonite**.**

Supplementary Table 2. Molality of CO_2 in analyzed fluids.

Supplementary Table 3. Deep Earth Water thermodynamic modelling of aqueous fluids at the investigated experimental conditions.

Supplementary Table 4. Conventional thermodynamic modelling of graphite-saturated fluids (C–O–H system) at the investigated experimental conditions.

Supplementary Table 5. Nano-SIMS analyses of graphite and aragonite in sample COH92.

Supplementary Table 6: LA-ICP-MS analyses of graphite and aragonite.

Supplementary Table 7: Parameters of Equation 6.

Supplementary Table 8: Thermal decomposition of oxalic acid di-hydrate and retrieval of the mass-bias QMS correction.

Supplementary Table 9: LA-ICP-MS analysis of standard carbonates.

Supplementary Table 10: Bulk analyses of labeled reagent and standard carbonates.

Supplementary Table 1: Run table of the experiments performed at 3 GPa–700°C and analyses of volatiles and of bulk after-run aragonite.

run	runtime (h)	buffer (double capsule)	starting materials	$Ca^{13}CO_3$ µmol	graphite µmol	$Ca^{13}CO_3$ / graphite	run products (+fluid)	total volatiles µmol	H_2O	CO_2	CH_4	CO_2 mol% ^{13}C	$X\,CO_2$ [a]	bulk aragonite mol% ^{13}C [c]	CO_2/ $Ca^{13}CO_3$
COH89	72	fs+mt+coe	$Ca^{13}CO_3 + H_2O$	55.6	-	-	arag ± cc	62.95	59.50	0.16	0.07	27.1	0.001[b]	n.m.	0.003
COH99	72	$Re+ReO_2$	$Ca^{13}CO_3 + H_2O$	52.6	-	-	arag ± cc	36.32	31.30	0.14	0.05	42.8	0.002[b]	n.m.	0.003
COH82	72	fs+mt+coe	$Ca^{13}CO_3$ + Cl-bear. $Zr(OH)_4$[d] + H_2O	91.0	-	-	arag + bdy ± cc	57.84	50.31	2.32	0.05	84.9	0.038[b,d]	n.m.	0.025
COH83	72	fs+mt+coe	$Ca^{13}CO_3$ + Cl-bear. $Zr(OH)_4$[e] + H_2O	52.4	-	-	arag + bdy ± cc	22.69	18.87	0.97	0.02	86.4	0.042[b,d]	n.m.	0.018
COH88	72	fs+mt+coe	graphite + H_2O	-	328.8	-	graph	14.93	6.58	4.81	0.05	1.43	0.422	-	-
COH96	240	fs+mt+coe	graphite + H_2O	-	331.3	-	graph	20.72	10.37	5.32	0.00	1.11	0.339	-	-
COH170	0.24	fs+mt+coe	graphite + $Ca^{13}CO_3 + H_2O$	10.2	219.8	0.044	graph + arag ± cc	30.29	26.21	0.70	0.10	62.8	0.026	96.1	0.069
COH186	0.24	fs+mt+coe	graphite + $Ca^{13}CO_3 + H_2O$	29.6	194.0	0.132	graph + arag ± cc	14.35	11.69	0.34	0.00	52.9	0.028	97.2	0.012
COH106	2.4	fs+mt+coe	grapiteh + $Ca^{13}CO_3 + H_2O$	24.9	183.2	0.120	graph + arag ± cc	21.10	18.06	0.71	0.01	80.5	0.038	95.8	0.028
COH98	24	fs+mt+coe	graphite + $Ca^{13}CO_3 + H_2O$	29.5	174.0	0.145	graph + arag ± cc	22.94	17.88	2.51	0.02	88.4	0.123	88.1	0.085
COH92	72	fs+mt+coe	graphite + $Ca^{13}CO_3 + H_2O$	28.3	188.1	0.131	graph + arag ± cc	28.44	21.28	3.93	0.02	81.7	0.156	89	0.139
COH97	240	fs+mt+coe	graphite + $Ca^{13}CO_3 + H_2O$	29.2	190.6	0.133	graph + arag ± cc	26.53	18.37	3.66	b.d.l.	82.2	0.166	82.9	0.125
COH171	0.24	fs+mt+coe	$Ca^{13}CO_3$ + OAD (0.3 mg)	37.8	-	-	arag ± cc	7.56	1.74	3.47	0.00	84.1	0.666	83.8	0.092
COH178	72	fs+mt+coe	$Ca^{13}CO_3$ + OAD (0.3 mg)	40.2	-	-	arag ± cc	11.07	3.89	5.27	0.04	79.9	0.575	80.8	0.131
COH184	72	fs+mt+coe	$Ca^{13}CO_3$ + OAD (0.3 mg)	29.9	-	-	arag ± cc	11.02	4.48	2.82	0.00	84.3	0.386	86.7	0.094
COH185	72	fs+mt+coe	$Ca^{13}CO_3$ + OAD (2 mg)	29.8	-	-	arag ± cc	41.52	17.08	17.99	0.05	54.7	0.513	55.3	0.604

a: $X\,CO_2 = CO_2/(H_2O+CO_2)_{molar}$

b: considering only $^{13}CO_2$

c: total decomposition by HCl + QMS analysis of evolved CO_2

d: chlorine in $Zr(OH)_4$ = 0.6 wt%; calculated final fluid Cl concentration = 0.89 mol Cl/kgH_2O

e: chlorine in $Zr(OH)_4$ = 0.6 wt%; calculated final fluid Cl concentration = 0.54 mol Cl/kgH_2O

n.m.: not measured

b.d.l.: below detection limit

fs: ferrosilite; mt: magnetite; coe:coesite; arag: aragonite; cc: calcite; graph: graphite; bdy: baddeleyite; OAD: oxalic acid di-hydrate

Supplementary Table 2: Molality of CO_2 in analyzed fluids.

Run	mol $^{13}CO_2$/ kgH_2O	mol $CO_{2(TOT)}$/ kgH_2O
COH89	0.041	-
COH99	0.108	-
COH82	2.169	-
COH83	2.460	-
COH88	-	39.99
COH96	-	28.16
COH170	-	1.482
COH186	-	1.617
COH106	-	2.167
COH98	-	7.794
COH92	-	10.26
COH97	-	11.05
COH171	-	110.6
COH178	-	75.25
COH184	-	34.86
COH185	-	58.46

$CO_{2(TOT)} = {}^{12}CO_2 + {}^{13}CO_2$

Supplementary Table 3: Deep Earth Water thermodynamic modelling of aqueous fluids at the investigated experimental conditions.

saturated phases	aragonite		graphite		graphite+aragonite
P (GPa)	3		3		3
T (°C)	700		700		700
$\log(f\,H_2/1\ \mathrm{bar})$	2.195		2.195		2.195
pH	5.090		2.210		4.200
$X\,CO_2=[CO_{2(AQ)}/(H_2O+CO_{2(AQ)}]$	0.0002		0.305		0.284
C bulk solubility (molality)	0.1205		24.40		24.05
predicted species (decreasing molality)		*predicted species (decreasing molality)*		*predicted species (decreasing molality)*	
OH-	1.12E-01	CO2(AQ)	2.44E+01	CO2(AQ)	2.20E+01
CA(HCO3)+	8.94E-02	H+	6.78E-03	CA(HCO3)+	8.90E-01
CA(OH)+	1.37E-02	CO(AQ)	5.67E-03	HCO3-	8.07E-01
CA(HCOO)+	1.10E-02	HCO3-	5.39E-03	HCOO-	2.01E-01
CO2(AQ)	8.80E-03	HCOOH	4.15E-03	CA(HCOO)+	1.49E-01
HCO3-	6.24E-03	HCOO-	1.32E-03	OH-	1.40E-02
CACO3(AQ)	3.50E-03	CH4(AQ)	3.19E-04	CO3--	8.95E-03
CA++	2.97E-03	H2(AQ)	1.77E-04	CO(AQ)	5.57E-03
HCOO-	1.15E-03	OH-	8.98E-05	HCOOH	4.00E-03
CO3--	2.46E-04	CH3COOH	3.67E-05	CACO3(AQ)	3.50E-03
H2(AQ)	1.77E-04	CH3CH2COO-	7.38E-07	CA++	6.42E-04
H+	1.12E-05	METHANOL(AQ)	7.20E-07	CH4(AQ)	3.19E-04
CO(AQ)	3.95E-06	ETHANOL(AQ)	3.00E-07	H2(AQ)	1.77E-04
HCOOH	3.84E-06	CH3COO-	2.24E-07	CA(OH)+	1.31E-04
CH4(AQ)	1.67E-07	CO3--	1.45E-07	CH3CH2COO-	1.13E-04
METHANOL(AQ)	5.02E-10	ETHANE(AQ)	1.17E-07	H+	1.12E-04
CH3COO-	1.03E-10	CH3CH2COOH	1.06E-07	CH3COOH	3.54E-05
CH3COOH	1.79E-11	ETHYLENE(AQ)	4.48E-09	CH3COO-	3.42E-05
CH3CH2COO-	1.77E-13	PROPANE(AQ)	8.26E-11	METHANOL(AQ)	7.07E-07
ETHANOL(AQ)	1.10E-13	O2(AQ)	1.25E-20	ETHANOL(AQ)	2.94E-07
ETHANE(AQ)	3.21E-14	PROPANOL	5.76E-25	ETHANE(AQ)	1.17E-07
ETHYLENE(AQ)	1.23E-15			CH3CH2COOH	1.02E-07
CH3CH2COOH	2.70E-17			ETHYLENE(AQ)	4.48E-09
O2(AQ)	2.20E-20			PROPANE(AQ)	8.26E-11
PROPANE(AQ)	1.20E-20			O2(AQ)	1.20E-20
PROPANOL	1.107E-34			PROPANOL	5.652E-25
abundances among carbon-bearing species					
% $Ca(HCO_3)^+$	74.27		-		3.70
% $CO_{2(AQ)}$	7.31		99.93		91.40

Supplementary Table 4: Conventional thermodynamic modelling of graphite-saturated fluids (C–O–H system) at the investigated experimental conditions.

P (GPa), T (° C)	3, 700
buffer	ferrosilite + magnetite + coesite
log(fO$_2$/1 bar) outer capsule[a]	-13.21
log(fH$_2$/1 bar) outer capsule = inner capsule[b]	2.195
log(fO$_2$/1 bar) inner capsule[c]	-13.36
XCO$_2$[c]	0.303
log(fO$_2$/1 bar)^{FMQ,d}	-13.97
ΔFMQ[e] inner capsule	+0.61

[a]log(fO$_2$/1 bar) of the buffering assemblage 3 $FeSiO_3$ + 0.5 O_2 = Fe_3O_4 + 3 SiO_2 (coesite), retrieved using the Perple_X package and the hp02ver.dat database.
[b] retrieved using the routine "fluids" of the Perple_X package (H–O HSMRK/MRK hybrid EoS).
[c]retrieved on the basis of constant log(fH$_2$/1 bar) using the routine "fluids" of the Perple_X package (GCOH-fluid MRK).
[d]log(fO$_2$/1 bar) of the metastable assemblage 3 Fe_2SiO_4 + O_2 = 2 Fe_3O_4 + 3 SiO_2 (quartz).
[e] ΔFMQ = log(fO$_2$/1 bar)^{sample} – log(fO$_2$/1 bar)^{FMQ}

Supplementary Table 5: Nano-SIMS analyses of graphite and aragonite in sample COH92.

Filename	^{13}C mol%	2 sigma
graphite		
analysis #1	1.119	0.006
analysis #2	1.141	0.003
analysis #3	1.171	0.003
analysis #4	1.181	0.003
aragonite		
analysis #1	4.101	0.006
analysis #2	8.81	0.04
analysis #3	10.12	0.04
analysis #4	13.04	0.05
analysis #5	20.3	0.2
analysis #6	24.0	0.8
analysis #7	27.3	0.2
analysis #8	28.6	0.3
analysis #9	29.1	0.2
analysis #10	31	1
analysis #11	31.6	0.1
analysis #12	32.4	0.7
analysis #13	41.4	0.8
analysis #14	41.5	0.7
analysis #15	84.0	0.8

Supplementary Table 6: LA-ICP-MS analyses of graphite and a

	^{13}C mol%	2 sigma
graphite		
COH106_1	1.4	0.2
COH106_2	1.5	0.2
COH106_3	1.2	0.1
COH98_1	1.2	0.1
COH98_2	1.1	0.1
COH92_1	1.1	0.1
aragonite		
COH106_1	65.2	0.7
COH106_2	71.5	0.7
COH106_3	70.5	0.7
COH106_4	67.8	0.7
COH106_5	70.6	0.7
COH106_6	55.5	0.6
COH106_7	58.9	0.6
COH106_8	76.9	0.8
COH106_9	74.9	0.7
COH106_10	76.4	0.8
COH106_11	58.8	0.6
COH106_12	59.0	0.6
COH106_13	81.6	0.8
COH106_14	71.6	0.7
COH106_15	71.9	0.7
COH98_1	45.5	0.5
COH98_2	45.3	0.5
COH98_3	46.2	0.5
COH98_4	47.0	0.5
COH92_1	36.2	0.4
COH92_2	27.6	0.3
COH92_3	38.7	0.4
COH92_4	38.6	0.4
COH92_5	42.0	0.4
COH92_6	37.9	0.4
COH92_7	38.4	0.4
COH92_8	43.7	0.4
COH92_9	49.8	0.5
COH92_10	41.0	0.4
COH92_11	45.9	0.5
COH97_1	36.5	0.4
COH97_2	98	1
COH97_3	88.9	0.9
COH97_4	55.4	0.6
COH97_5	38.0	0.4
COH97_6	73.7	0.7
COH97_7	60.5	0.6
COH97_8	53.1	0.5
COH97_9	52.2	0.5
COH97_10	57.2	0.6
COH97_11	63.0	0.6

Supplementary Table 7: Parameters of Equation 6.

Independent variable	Parameter estimate	sigma
1	4.71676	0.0507989
WAR	-2.87021	0.0494956
ΔFMQ	-3.00212	0.163084
ΔFMQ^2	2.15874	0.238246
ΔFMQ^3	-2.08921	0.118716
ΔFMQ x WAR	0.294843	0.0586424
WAR^2	1.02176	0.0267483
WAR^3	-0.12774	0.00443806
ΔFMQ^2 x WAR	-1.02538	0.0513343
WAR^{-2}	0.0030557	0.00010255

WAR=water/aragonite molar ratio.

$\Delta FMQ = \log(fO_2/1\ \text{bar})^{sample} - \log(fO_2/1\ \text{bar})^{FMQ}$

Supplementary Table 8: Thermal decomposition of oxalic acid di-hydrate and retrieval of the mass-bias QMS correction factor.

	^{12}C-OAD mg	^{13}C-OAD mg	predicted $^{13}C/^{12}C$	predicted ^{13}C abundance mol%	integrated peak channel 44	integrated peak channel 45	ratio 45/44	ratio $45_{corr}^{a}/44$	^{13}C abundance %
regular OAD	3.6	0	0.013	1.25	5.53E-06	7.02E-08	0.013	-	1.29
labeled ^{13}C-OAD	0	9.91	191.283	99.5	2.86E-07	5.47E-05	191.283	-	99.5
mix 1	5.55	5.11	0.922	48.0	7.45E-06	6.38E-06	0.856	0.881	46.9
mix 2	7.52	1.53	0.214	17.6	8.79E-06	2.40E-06	0.273	0.281	22.0
mix 3	0.72	9.06	11.729	92.1	3.92E-06	4.45E-05	11.364	11.705	92.1
test_AA8	0.56	0.86	1.52	60.4	0.00	0.00	1.35	1.39	58.1

OAD: oxalic acid di-hydrate

a: using correction factor 1.03 for channel 45, corresponding to a regression line through mix1–mix2–mix3 with R^2=0.99993

Supplementary Table 9: LA-ICP-MS of standard carbonates.

	^{13}C mol%	2 sigma
natural calcite		
natural_calcite_1	1.07	0.05
natural_calcite_2	1.09	0.05
natural_calcite_3	1.15	0.06
natural_calcite_4	1.12	0.06
natural_calcite_5	1.10	0.05
synthetic calcite		
synthetitc_calcite_1	43.9	0.4
synthetitc_calcite_2	43.7	0.4
synthetitc_calcite_3	44.4	0.4

synthetic calcite is precipitated from mixed ^{12}C–^{13}C Na_2CO_3 solutions.

Supplementary Table 10: Bulk analyses of labelled reagent and standard carbonates.

	integrated peak channel 44	integrated peak channel 45	ratio 45/44	ratio $45_{corr}^{a}/44$	^{13}C abundance mol%
synthetic ^{13}C calcite	6.83E-08	1.18E-05	173	178	99.4
precipitated (^{12}C, ^{13}C) synthetitc calcite	4.45E-06	3.36E-06	0.755	0.778	43.8
natural-abundance calcite	5.62E-06	6.22E-08	0.0111	0.0114	1.13

a: using correction factor 1.03 for the integrated peak of channel 45.
